# ESMRMB 2025 focus topic: cycle of quality—from concept to clinical and scientific impact

**DOI:** 10.1007/s10334-025-01272-0

**Published:** 2025-06-14

**Authors:** Thomas Küstner, Roy Haast, Nikos Priovoulos, Patricia Clement, Daniel Pinto dos Santos

**Affiliations:** 1 Medical Image and Data Analysis (MIDAS.Lab), University Hospital of Tübingen, Tübingen, Germany; 2 Aix Marseille Univ, CNRS, CRMBM, Marseille, France; 3 APHM, Hôpital Universitaire Timone, CEMEREM, Marseille, France; 4 Wellcome Centre for Integrative Neuroimaging, FMRIB, Nuffield Department of Clinical Neurosciences, University of Oxford, Oxford, UK; 5 Department of Medical Imaging, Ghent University Hospital, Ghent, Belgium; 6 Department of Diagnostic Sciences, Ghent University, Ghent, Belgium; 7 Department of Radiology, University Hospital Mainz, Mainz, Germany

## Introduction

Magnetic resonance imaging (MRI) is increasingly positioned as both a routine clinical tool and a measurement instrument in biomedical research. In recent years, the discussion of “quality” in MRI has evolved beyond the technical performance of hardware or the clarity of images. It now encompasses a comprehensive ecosystem spanning scanner operation, data integrity, harmonization across sites and platforms, algorithmic robustness—particularly in artificial intelligence (AI)—and the reproducibility and dissemination of research outputs. This year’s ESMRMB conference takes on this complexity under the focus topic “The Cycle of Quality,” aiming to dissect and connect each element contributing to high-quality MRI, as a dynamic, interconnected process. Across the plenary and four educational sessions, the speakers explore how quality manifests at every stage of the MRI cycle: from acquisition and analysis, to algorithmic design, to harmonization, and ultimately, to research dissemination. 

## Plenary: good for what? Defining quality from a practical perspective

The plenary session provides a conceptual foundation for the quality cycle, emphasizing that quality must always be contextualized by its purpose. In her presentation, **Martina Callaghan** (*“Beyond accuracy: Towards high-quality MR biomarkers”*) draws a picture of moving past simplistic one-dimensional metrics, and instead considers biological relevance, reproducibility across populations and platforms, and long-term robustness of biomarkers. She will describe the developments of biomarkers with well-characterized confounds and uncertainty, as well as the infrastructure needed for reproducibility across vendor ecosystems.

Complementing this, **Gaël Varoquaux** (*“Quality control of AI in MRI: From evidence to evaluation”*) addresses the increasing integration of AI into MRI workflows. He highlights the critical lack of standardized evaluation frameworks for AI methods, showcasing that reproducibility, interpretability, and generalizability should be considered part of quality assurance. AI models must be developed with the same rigor expected of traditional imaging biomarkers, grounded in sound methodology and validated across diverse, real-world datasets.

Together, these talks emphasize that “quality” is a purpose-driven attribute: what counts as high-quality for biomarker discovery may differ from what is needed in clinical triage or time-critical scenarios, but all applications benefit from clearer definitions and reproducible standards.

## Educational 1: harmonizing research

MRI harmonization remains a cornerstone in the pursuit of reproducible science. The first educational session explores how variability across scanners, vendors, and protocols can confound findings and limit the generalizability of research.

**Qingping Chen** (*“Scanner variability in MRI: Challenges and solutions for harmonization”*) provides an in-depth technical analysis of why standardizing MRI protocols is so difficult in practice. Despite the use of vendor-supplied sequences, heterogeneity in gradient strengths, slew rates, and reconstruction filters leads to inconsistent outputs. Chen presents a compelling harmonization framework that integrates Pulseq, Gadgetron, and an open-source, automated post-processing pipeline. This vendor-independent quality assurance protocol represents a critical step toward platform-neutral standardization.

**Shaun Warrington** (*“Harmonizing statistical models: Reproducibility and reliability”*) shifts the focus from prospective harmonization to retrospective harmonization based on statistical modeling. His talk underlines how statistical corrections—whether in image or feature space—can address site effects, but must themselves be rigorously validated. He introduces site predictability, phantom assessments, and traveling-heads studies (like ON-Harmony) as tools for benchmarking harmonization success. Warrington argues that harmonization is not just a preprocessing step, but a modeling philosophy aimed at separating biological variability from technical noise.

Finally, **Bertrand Thirion** (*“AI and Harmonization: Standardization for robust algorithms?”*) tackles the interaction between AI and harmonization, asking whether harmonization should precede AI modeling or be embedded within it. The importance of model robustness and transferability is highlighted, and harmonization practices that are co-designed with AI development are advocated—an approach that ensures learned representations are not just locally accurate but globally generalizable.

## Educational 2: quantitative MRI and its confoundings

Quantitative MRI (qMRI) offers great potential for objective biomarkers but is notoriously sensitive to confounding factors. This session addresses the technical and translational challenges of ensuring reliable and reproducible qMRI measurements.

**Siawoosh Mohammadi** (*“Quantitative MRI: Understanding and mitigating confounding factors”*) introduces the multifactorial nature of qMRI confounds—ranging from physiological noise and scanner instabilities to the specific choice of fitting models and parameters. He outlines strategies to quantify and correct for these influences, noting the importance of integrating correction steps into standardized pipelines.

Building on this, **Sebastian Weingartner** (*“Quality Matters: Assessing reliability in quantitative MRI”*) provides a framework for validating qMRI techniques. His talk highlights the distinction between technical performance (bias, noise, repeatability) and clinical performance, and urges researchers to adopt structured validation designs involving phantoms, reference standards, and transparent error reporting. By doing so, Weingartner argues, the translational “valley of death” for promising qMRI methods can be narrowed.

**Amy McDowell** (*“Clinical Applications of qMRI: Ensuring Accuracy and Reproducibility”*) provides a clinical perspective on qMRI. She calls for a metrological foundation for qMRI in routine care, stressing that quantitative values must be interpretable, contextualized (e.g., with normative ranges), and traceable to validated standards. She compares existing phantom-based calibrations with emerging AI-based harmonization strategies, and advocates for deeper engagement of hospital physicists in ensuring measurement reliability at the point of care.

## Educational 3: quality assessment and control in the clinical setting

This session addresses the challenges and innovations in maintaining quality within real-world clinical environments. Together, these talks underscore that maintaining quality in clinical MRI is not a static task but a dynamic process that must evolve with changing technologies, operational models, and clinical demands. Whether through workflow optimization, the adoption of meaningful image metrics or quality governance for remote scanning, the session offers actionable insights for making quality an embedded, adaptive feature of modern clinical MRI.

**Susie Huang** (*“From acquisition to reporting: Ensuring quality and efficiency in MRI workflows”*) offers a pragmatic view of how quality assurance must span the entire imaging chain. She maps out critical quality checkpoints, from protocol setup and staff training to post-processing and reporting, highlighting the need for efficiency without sacrificing reliability.

**Simone Busoni** (*“Beyond SNR: Advanced metrics for MRI quality control”*) encourages to adopt more nuanced and informative quality metrics. Task-based evaluations, artifact quantification, and visual integrity scores as alternatives or supplements to traditional measures like signal-to-noise ratio (SNR) are presented. These metrics can better capture the diagnostic utility and robustness of images, especially in complex clinical cases or under constrained conditions such as remote scanning.

**Anton Quinsten** (*“Quality aspects in remote scanning”*) addresses the emerging field of remote and tele-operated MRI, which is reshaping how and where scans are performed. As remote scanning becomes increasingly viable—especially in underserved or rural areas—it brings both opportunities and challenges. Quinsten discusses the specific quality risks associated with decentralized scanning, such as variable scanner calibration, protocol drift, inconsistent operator expertise, and difficulties in real-time supervision. He proposes strategies to mitigate these risks, including centralized protocol management, remote training, and support, automated QA dashboards, and real-time performance monitoring. His presentation highlights the urgent need for tailored quality frameworks that support safe and effective remote imaging without compromising diagnostic reliability.

## Educational 4: quality of dissemination in research outputs

While much of the quality discourse in MRI centers on image acquisition, algorithm design, and validation methods, the final step in the cycle—how we share and publish our research—is just as critical. Scientific dissemination is not merely the endpoint of a project; it is a core mechanism by which knowledge is evaluated, reused, and translated into clinical or technological impact. Yet, despite advances in imaging and data science, dissemination practices often lag behind, with limited transparency, reproducibility, or accessibility. This session focuses on the dimension of quality: how MRI research is communicated, credited, and archived. It raises important questions about authorship practices, data availability, code sharing, reproducibility, and the role of journals and reviewers in enforcing quality standards.

**Nikola Stikov** (*“Publishing with purpose: Collaborating for high-quality MRI research”*) argues for a cultural shift toward more collaborative, transparent, and reproducible publishing. He shows the importance of openly shared code, data, and protocols, and calls for improved reporting standards and multi-disciplinary authorship models.

**Stefano Moia** (*“From submission to citation: Ensuring quality in published MRI research”*) explores how our heuristics and expectations about journals, peer reviewers, and indexing systems can sometimes trick us in understanding the quality of the scientific dissemination. He will then discuss how open and reproducible science practices in manuscript writing, reporting, and reviewing (e.g. reproducibility checklists, open reviews, metadata) can improve the FAIRness and soundness of scientific work.

## Conclusion

The “Cycle of Quality” is not a linear checklist—it is a loop of interconnected efforts spanning acquisition, processing, modeling, application, and communication. As shown across the plenary and educational sessions, ensuring quality in MRI requires more than improved technology: it calls for cultural alignment, shared infrastructure, and a collective commitment to transparency and reproducibility. Whether it is through harmonized imaging protocols, robust statistical models, reliable qMRI validation, or trustworthy AI deployment, the field is increasingly equipped to meet these challenges. The ESMRMB 2025 conference wants to highlight that achieving high-quality MRI depends not only on technological breakthroughs, but equally on shared community standards and collaborative practices. 

## Data Availability

We do not analyse or generate any datasets, because our commentary describes a scientific meeting.

